# Supplementary material for: Primary Healthcare Physicians’ Insufficient Knowledge Is Associated with Antibiotic Overprescribing for Acute Upper Respiratory Tract Infections in China: A Cross-Sectional Study
Source: Antibiotics (Basel). 2024 Sep 26;13(10):923. doi: 10.3390/antibiotics13100923 (PMC11505141; doi:10.3390/antibiotics13100923)
Supplement: Supplementary file 1 [file antibiotics-13-00923-s001.zip › antibiotics-3206504-supplementary.pdf]

Table S1 Gross Domestic Product (GDP) ranking of each prefecture-level region of Shaanxi Province in 2020.

Prefecture-level cities	GDP (billions Chinese Yuan)	Ranking of GDP
Xi’an	932.119	1
Yulin	413.628	2
Baoji	222.725	3
Xianyang	219.533	4
Weinan	182.847	5
Yan’an	166.389	6
Hanzhong	154.759	7
Ankang	118.206	8
Shangluo	83.721	9
Tongchuan	35.472	10

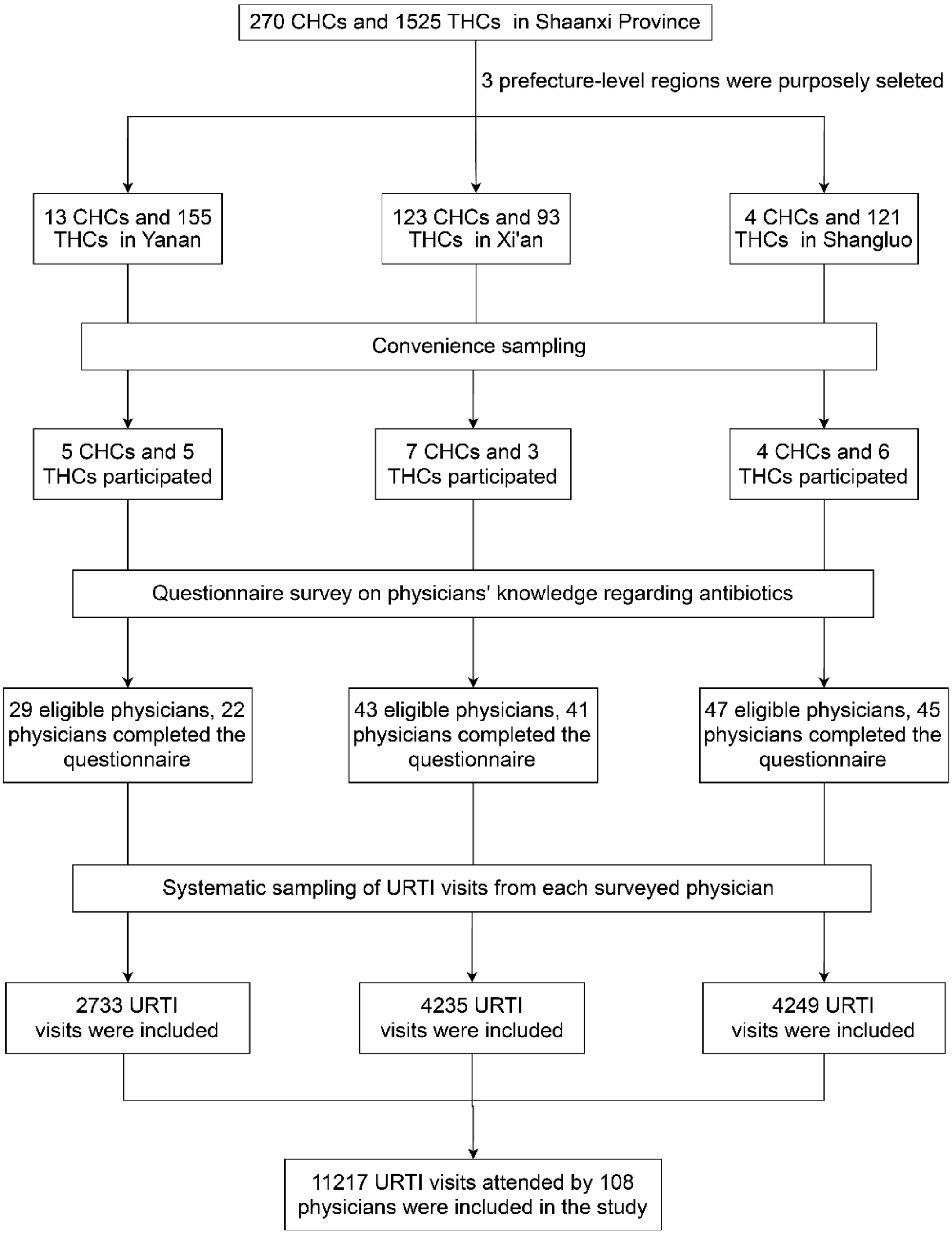

Figure S1 Sampling procedures.

Note: CHCs: Community healthcare centers. THCs: Township healthcare centers

Table S2 Characteristics of the sampled acute URTI visits.

	CHCs	THCs	Total
Total visits	5477(48.8)	5740(51.2)	11217
Patient Gender			
Male	2503(45.7)	2929(51)	5432(48.4)
Female	2974(54.3)	2811(49)	5785(51.6)
Patient age	35.9 ± 20.2	37.2 ± 21.1	36.5 ± 20.7
Patient age group			
≤ 16 years	1215(22.2)	1339(23.3)	2554(22.8)
17 - 45 years	2334(42.6)	2098(36.6)	4432(39.5)
46 - 65 years	1474(26.9)	1722(30.0)	3196(28.5)
66 - 80 years	454(8.3)	581(10.1)	1035(9.2)
Diagnoses			
J00	44(0.8)	350(6.1)	394(3.5)
J01	4(<0.1)	11(0.2)	15(0.1)
J02	680(12.4)	965(16.8)	1645(14.7)
J03	242(4.4)	319(5.6)	561(5.0)
J04	62(1.1)	94(1.6)	156(1.4)
J05	0	0	0
J06	4445(81.2)	4001(69.7)	8446(75.3)
Payment method			
Fully out-of-pocket	2946(53.8)	2181(38.0)	5127(45.7)
With insurance co-payment	2531(46.2)	3559(62.0)	6090(54.3)

Notes: Data are n (%). CHCs: Community healthcare centers. THCs: Township healthcare centers.
